# EasyCellType: marker-based cell-type annotation by automatically querying multiple databases

**DOI:** 10.1093/bioadv/vbad029

**Published:** 2023-03-24

**Authors:** Ruoxing Li, Jianjun Zhang, Ziyi Li

**Affiliations:** Department of Biostatistics and Data Science, The University of Texas Health Science Center at Houston, Houston, TX 77030, USA; Department of Biostatistics, The University of Texas MD Anderson Cancer Center, Houston, TX 77030, USA; Division of Cancer Medicine, Department of Thoracic-Health & Neck Med Oncology, The University of Texas MD Anderson Cancer Center, Houston, TX 77030, USA; Department of Biostatistics, The University of Texas MD Anderson Cancer Center, Houston, TX 77030, USA

## Abstract

**Motivation:**

Cell label annotation is a challenging step in the analysis of single-cell RNA sequencing (scRNA-seq) data, especially for tissue types that are less commonly studied. The accumulation of scRNA-seq studies and biological knowledge leads to several well-maintained cell marker databases. Manually examining the cell marker lists against these databases can be difficult due to the large amount of available information. Additionally, simply overlapping the two lists without considering gene ranking might lead to unreliable results. Thus, an automated method with careful statistical testing is needed to facilitate the usage of these databases.

**Results:**

We develop a user-friendly computational tool, EasyCellType, which automatically checks an input marker list obtained by differential expression analysis against the databases and provides annotation recommendations in graphical outcomes. The package provides two statistical tests, gene set enrichment analysis and a modified version of Fisher’s exact test, as well as customized database and tissue type choices. We also provide an interactive shiny application to annotate cells in a user-friendly graphical user interface. The simulation study and real-data applications demonstrate favorable results by the proposed method.

**Availability and implementation:**

https://biostatistics.mdanderson.org/shinyapps/EasyCellType/; https://bioconductor.org/packages/devel/bioc/html/EasyCellType.html.

**Supplementary information:**

Supplementary data are available at *Bioinformatics Advances* online.

## 1 Introduction

Single-cell RNA sequencing (scRNA-seq) technology was first published in 2009 (Tang *et al.*, 2009) and soon became an effective tool for sequencing the transcriptomes of different organisms at a single-cell level (Lee *et al.*, 2020; Li *et al.*, 2020; Potter, 2018). It has been widely used in studying the cellular heterogeneity of various tissue samples and identifying phenotype-associated cell groups (Lawson *et al.*, 2015; Lim *et al.*, 2020; Mathys *et al.*, 2019). It also provides exciting opportunities to dissect complex cell behaviors, such as lineage tracing (Frieda *et al.*, 2017) and cell–cell communication (Almet *et al.*, 2021; Kumar *et al.*, 2018).

Analyzing an scRNA-seq dataset typically starts from quality control and alignment (Kaminow *et al.*, 2021), batch effect correction (Chazarra-Gil *et al.*, 2021), dimension reduction (Zhou and Jin, 2020) and clustering (Kiselev *et al.*, 2017; Satija *et al.*, 2015), followed by a crucial step—cell annotation. In general, cells from scRNA-seq data are annotated mainly through two approaches: one is manual annotation based on the cluster-specific differentially expressed marker genes and the other is supervised cell annotation using existing datasets as the reference. The former approach can be labor intensive and time consuming. Moreover, it requires analyzers to have a good understanding of the potential cell types contained in collected scRNA-seq datasets as well as the corresponding cell-type-specific markers (Tran, 2022). Recognizing these challenges, a number of supervised annotation methods have also been developed to fully utilize the published scRNA-seq data as the training set and to predict the cell labels in the new dataset (Lin *et al.*, 2020; Ma and Pellegrini, 2020). However, the supervised method can be limited if the annotated data from the matched tissue types are not available, or the published data are from a different condition, e.g. healthy versus diseased (Sun *et al.*, 2022).

With the accumulation of scRNA-seq research, great efforts have been made to summarize the tissue-specific marker information, and a few comprehensive databases become available, e.g. CellMarker (Zhang *et al.*, 2019b), PanglaoDB (Franzén *et al.*, 2019) and Clustermole (Dolgalev, 2021), which provide useful information for cell-type identities. However, manually checking the cell-type-specific markers against these databases can be an impractical task due to the large amount of information. A few marker-based annotation methods have also been developed. CellAssign used a probabilistic model to annotate individual cells based on collected marker genes (Zhang *et al.*, 2019a). The method can achieve an accuracy of higher than 90% when well-understood marker genes exist. However, users need to derive their own marker gene sets for annotation usage, which may not be easy for scientists without biological expertise. A semi-supervised method, SCINA, is applicable to classify cell type for scRNA-seq, CyTOF/FACS and bulk RNA-seq data (Zhang *et al.*, 2019c), but a curated gene list is also required. SCSA by Cao *et al.* (2020) integrates the marker information from existing databases to assign cell types. SCSA uses a score annotation model to rank the overlapped genes between the differential signals and the databases. For the genes that are not included in the cell marker database, SCSA will perform gene ontology (GO) enrichment analysis to interpret the biological processes. When SCSA was first published, it only supported the clustering output from CellRanger or Seurat as its input. Now, although the clustering output from Scanpy or Scran is also supported, the applications are still limited. More recently, scGate (Andreatta *et al.*, 2022) and MACA (Xu *et al.*, 2022) were also developed based on marker databases. MACA is an scRNA-seq annotation tool that uses normalized confusion matrices. scGate can annotate not only scRNA-seq data but also other single-cell modalities (e.g. ATAC-seq). scGate and MACA have similar challenges as CellAssign that the cell types in the query dataset need to be pre-defined. Thus, they may not be friendly to less-studied tissues or diseased conditions.

To facilitate researchers to use different databases directly and conveniently, we develop a computational tool, EasyCellType, which can automatically examine the input marker lists obtained by differential expression analysis using existing methods, including but not limited to Seurat and SC3, and check against the available cell marker databases. We provide two quantification approaches to annotate cell types: gene set enrichment analysis (GSEA) and a modified Fisher’s exact test that takes statistical significance into consideration. We wrap our methods in a user-friendly R package. The package generates straightforward results including visualization of the test results across all clusters and the prediction rankings within each cluster, which help the researchers to understand the results and pick the optimal annotations. To enable users without much programming experience, we also developed an interactive shiny application with a graphical user interface (GUI) that can be launched at https://biostatistics.mdanderson.org/shinyapps/EasyCellType/. Our tools allow annotating cells from two species (human and mouse) and around 300 organs, including common (e.g. blood and brain) and rare tissue types (e.g. umbilical vein and renal glomerulus).

## 2 Methods

**Fig. 1.**
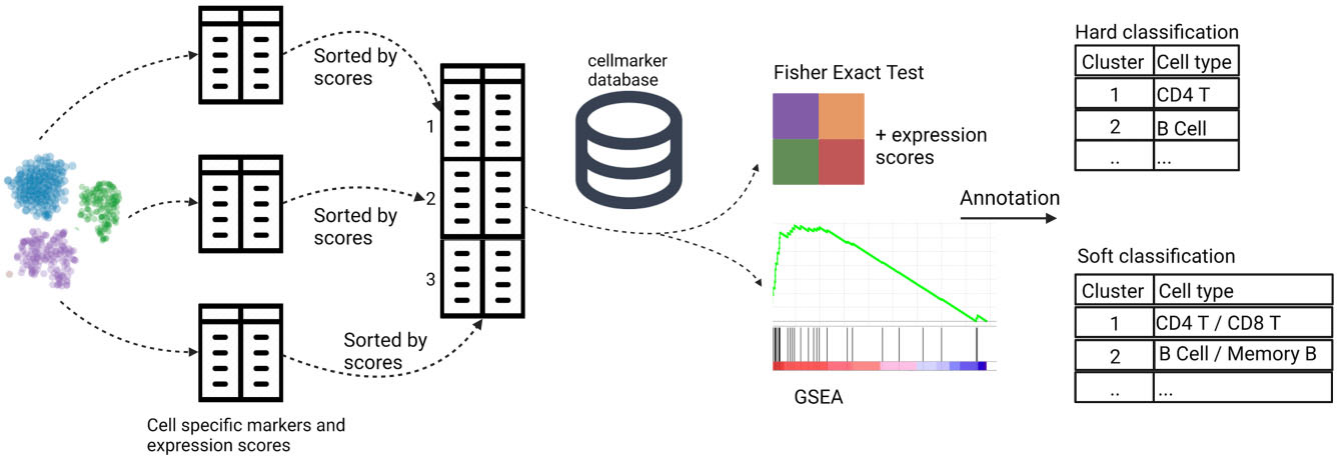
Schema of the package setup. scRNA-seq data are analyzed and generate set of markers with the differential expression score. Taking the clustered markers as input, user then can specify the database and tissue to run GSEA or Fisher’s exact test to annotate the group of cells. The annotation results will be presented in two ways: hard classification gives the most likely cell type for each cluster, and soft classification gives the several top possible cell types for each cluster

A schematic pipeline of the proposal is presented in Figure 1. After preprocessing (e.g. normalized, filtered and dimension reduced) and clustering of the scRNA-seq data, we expect users to first extract cell-type-specific differentially expressed markers for each cluster and sort the gene lists by their differential score (e.g. log fold change). We then compare the input marker list against existing databases (details in Section 2.1 below) using a statistical test, either GSEA or a modified Fisher’s exact test (details in Section 2.2 below). We also provide two possible ways to present the results, hard clustering and soft clustering, for the best-predicted cell type (‘hard’) or a list of ranked possible cell types (‘soft’). This allows users to pick the most interpretable results when other auxiliary information is available.

### 2.1 Database description

We include three publicly available cell marker databases in our method: CellMarker, PanglaoDB and Clustermole. To keep consistency with the databases, we provide functions to convert gene symbols to Entrez IDs which will be used in the annotation analysis.

Zhang *et al.* (2019b) manually reviewed 10 000 papers from PubMed related to single-cell analysis and cell marker experimental studies to build CellMarker database. The database includes 467 and 389 cell types for human and mouse, respectively. The database is freely available for download at http://bio-bigdata.hrbmu.edu.cn/CellMarker/.

The PanglaoDB is a web server designed for scientists to explore published scRNA-seq experiments from mouse and human. During the integration of scRNA-seq studies, to ensure reliable biological insights, cell markers were manually collected by querying thousands of papers and search engines (Franzén *et al.*, 2019). These markers consist of canonical markers and novel markers, the latter were used when canonical markers were ambiguous by examining expressed genes in the particular single-cell cluster. At the time of this publication, it includes 178 cell types both for human and mouse.

Clustermole (Dolgalev, 2021) is a pipeline that can process scRNA-seq data. It collects cell marker genes from seven sources: ARCHS4, CellMarker, MSigDB, PanglaoDB, SaVanT, TISSUES and xCell. We extract those genes with species information (human or mouse), resulting in 1694 cell types for human and 331 cell types for mouse.

Supplementary Figures S1 and S2 present how cell types and tissues are intersected among the three databases. Database from Clustermole contains most human cell types. No big difference is found regarding the number of mouse cell types among the three databases. Compared to the other two databases, Clustermole contains some unique cell types such as Abomasum and Krt4/13+. Comparing the cell-type classification from the same tissue type, the CellMarker database generally has a more detailed classification, while PanglaoDB has a broader lineage classification.

### 2.2 Algorithm

We make use of the GSEA algorithm to facilitate the cell-type annotation task. Originally, GSEA (Subramanian *et al.*, 2005) takes a set of genes sorted by their statistical importance and tests whether a priori-defined set of genes shows statistically significant associations with the phenotype. Details of the original algorithm are described in the Supplementary material. GSEA has been widely used in GO pathway analysis. Algorithms have been updated to facilitate other analysis procedures, such as FGSEA (Korotkevich *et al.*, 2019). Besides the GSEA software, multiple packages, such as ‘fgsea’ and ‘clusterProfiler’ (Wu *et al.*, 2021b) are available in R and ready for users to customize the analysis with different pathway choices. For our usage, given a list of marker genes obtained by differential expression analysis from any existing clustering software, we sort the marker genes by their expression scores (e.g. log 2 fold change). The priori-defined set of genes in GSEA methods will be replaced as the known cell-specific marker genes from the multiple marker databases. We will then compute the cell types that are significantly associated with the differentially expressed genes using the GSEA algorithm.

Although we believe GSEA can be powerful in cell-type annotation, in reality, when a researcher wants to annotate clustered cells, a generally used approach is to simply check what cell types are defined by the most frequent marker genes. We see the logic of this procedure as similar to the logic of testing a contingency table: determining the group for a categorical variable based on the collected prevalence frequencies. Thus, we provide a modified version of Fisher’s exact test to reproduce the procedure described here. Fisher’s exact test is a straightforward way to assess the significance of contingency between two kinds of classifications. Extending this method, given a vector of clustered markers and a vector of labeled genes, in each cluster we examine the significance of the association between each marker and labeled genes. The estimated significance is then adjusted by Benjamini–Hochberg procedure to account for the multiple hypothesis testing. Since the vector of markers typically obtained by scRNA-seq data analysis comes with a statistical score, such as log fold change, showing expression significance, we add one more step by sorting the associations in each cluster based on the markers’ expression score, and select the tops as annotation results.

We present the annotation results in two ways: hard classification and soft classification to suit different needs of the users. Hard classification gives the most significant cell type for each cluster. In contrast, soft classification gives the top five significant cell types received from the test. Hard classification may be suitable for annotating tissues that are well studied, such as blood and brain, for which comprehensive lists of cell markers are available. In these cases, the hard classification results can provide an accurate set of cell labels. Soft classification can be more feasible if the tissue of interest is from a different condition or less-commonly studied tissues, such as brain samples from autism patients. Users can choose the best interpretable cell labels from the provided lists to achieve more accurate results.

### 2.3 Package and shiny app

Our R package is freely available at Bioconductor (‘EasyCellType’) or from GitHub (https://github.com/rx-li/EasyCellType). It is straightforward to conduct cell-type annotation using our package. The input is a data frame containing Entrez IDs of marker genes, cluster number as well as the corresponding expression score. If the input data use gene symbols, we provide a function to automatically convert the gene symbols to Entrez IDs. Users need to specify the database, species and tissue (organ) for the annotation. Among all the three databases, we set the CellMarker database for default usage based on the simulation results in Section 3.3, if the studied tissue type is available. After getting the annotation results, functions are available to create bar plots for each cluster showing candidate cell types, as well as a dot plot summarizing the top five significant annotations for each cluster.

**Fig. 2.**
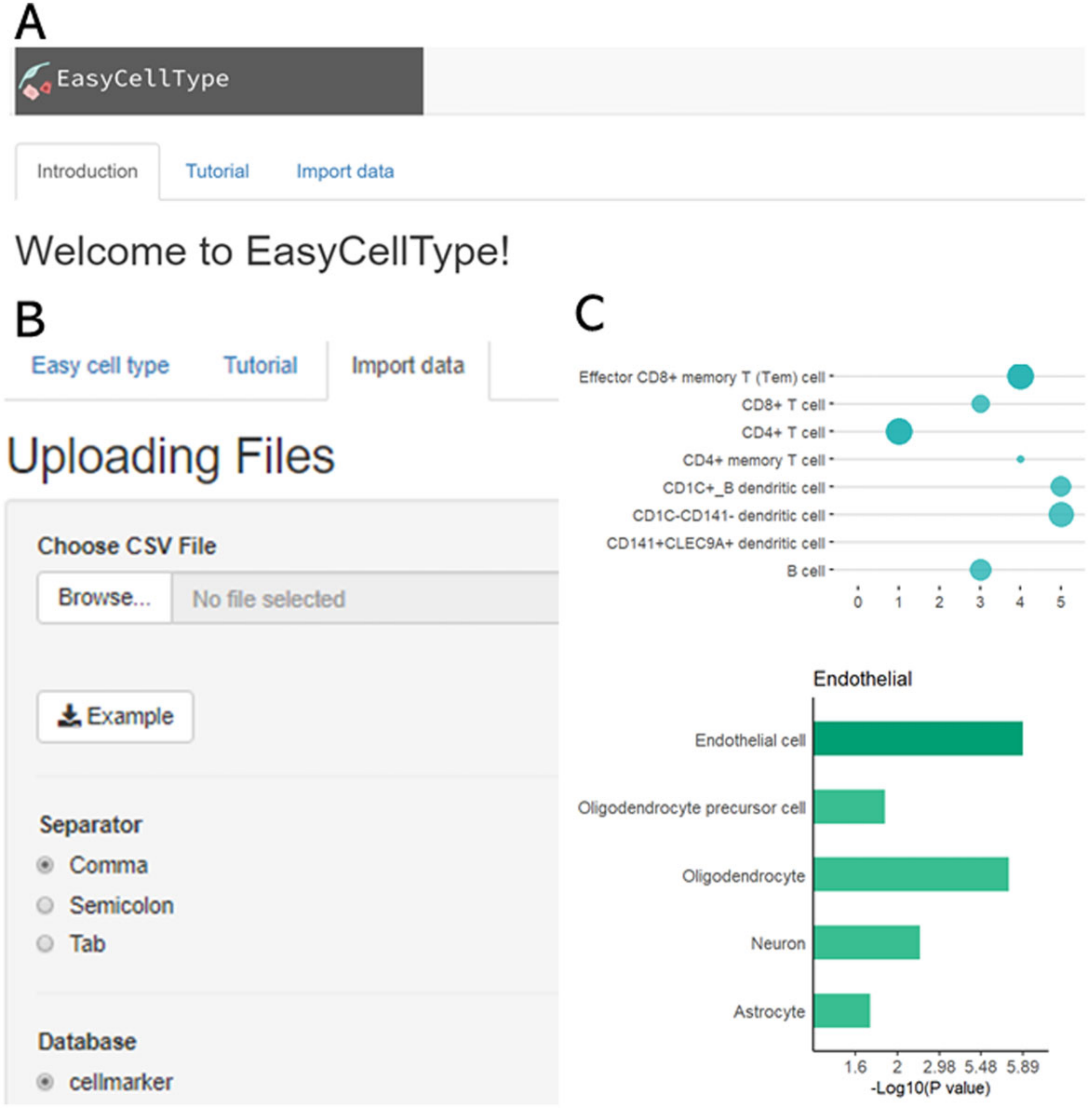
Overview of the shiny application. This application was designed for users to straightforwardly launch and use the EasyCellType package. (**A**) The navigation bar of the application. Three pages are included in the web: introduction, tutorial and input panel. (**B**) Uploading page. Users would need to upload a CSV file containing marker gene, cluster information and expression score (e.g. log 2 fold change) to run the analysis. There is an ‘Example’ file that can be downloaded and referred to. Database and species can be specified according to the file. Users can choose tissue (organ) type and test used for the annotation. If the user chooses to use GSEA for the analysis, the *P*-value and score type can also be specified. (**C**) The output of the application. After finishing the analysis, dot plots and bar plots will be created, and ready to be downloaded

For users that are not familiar with R coding, our method has also been implemented in an interactive shiny web freely available at https://biostatistics.mdanderson.org/shinyapps/EasyCellType/. Figure 2 presents screenshots of the GUI from the shiny app. A brief introduction of EasyCellType can be found on the first page (Fig. 2A). On the second page, we provide a detailed tutorial describing how to upload files and run analysis using the application. User can find the uploading panel on the third page (Fig. 2B). User needs to upload a CSV file containing marker genes in gene symbols (will be converted to Entrez IDs) or Entrez IDs, cluster numbers and differential testing score (e.g. log fold change), then specify database and species, and choose tissue types for the analysis. Dot plots and bar plots will be created and presented on the right side of the screen after finishing the analysis and will be ready to be downloaded (Fig. 2C).

## 3 Simulation study

To evaluate the annotation accuracy of the proposed methods, we design a series of simulation studies using the peripheral blood mononuclear cells (PBMC) data sequenced by the 10X platform (Zheng *et al.*, 2017). This dataset contains 32 738 genes from eight cell types: B cells, CD14 monocytes, CD56 NK cells, CD4 T helper cells, memory T cells, naive cytotoxic T cells, naive T cells and regulatory T cells. We randomly draw cells from the original scRNA-seq dataset to obtain the simulation data consisting of all eight cell types. The total number of cells is fixed at 1600, 2400 or 4000.

We convert gene symbols to Enterz IDs for the identified differential cluster-specific markers. We use CellMarker database as the default marker database, while more comparison results using different databases are discussed in Section 3.3. Tissue types containing keyword ‘blood’ are chosen as the organ in the database, including blood, peripheral blood, blood vessel, umbilical cord blood and venous blood. We then perform the GSEA and the modified version of Fisher’s exact test to annotate the cells. We check the annotation for each cell to calculate the accuracy. If a cell is same as the annotation for the cluster that it belongs to, we will say that the cell is annotated correctly. Specifically for soft classification, if any of the proposed labels are correct, we will say that the method annotates the cell correctly. The accuracy is defined as the number of cells with correct annotation divided by the number of total cells. For each combination of simulation settings, the presented results are summarized over 100 Monte Carlo simulation iterations.

### 3.1 Comparison with existing annotation tools

**Fig. 3.**
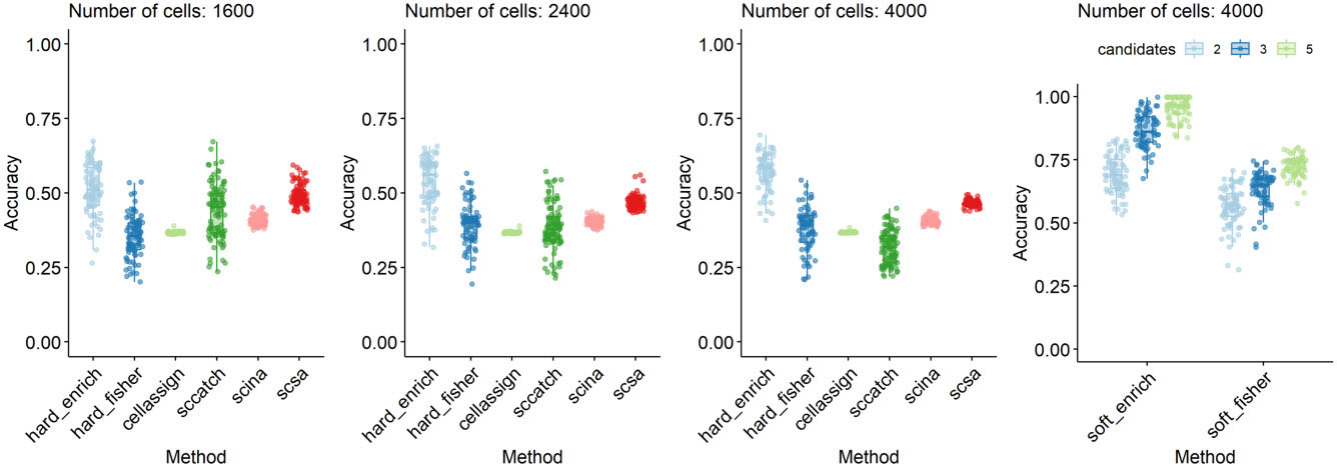
Accuracy of cell-type annotation under different cell number settings using the proposed method, CellAssign, scCATCH, SCINA and SCSA. ‘hard_enrich’ means that we use GSEA approach and hard classification. The same patterns are followed by other notations on the X-axis. (**A**) Sample datasets contained 1600 PBMCs. (**B**) Sample datasets contained 2400 PBMCs. (**C**) Sample datasets contained 4000 PBMCs. (**D**) The annotation accuracy of soft classifications using GSEA and fisher approaches with two, three and five cell-type predictions

We compare our methods with unsupervised annotation tools, CellAssign, scCATCH, SCINA and SCSA through a series of simulations with a different number of cells. The results are presented in Figure 3. For the proposed method, we use Seurat for clustering and identifying differentially expressed genes. For scCATCH, we use its default marker genes database. Tissue types are chosen as ‘blood related’ which is suggested on its website (https://github.com/ZJUFanLab/scCATCH/wiki/human_tissues): blood, peripheral blood, plasma, serum, umbilical cord blood and venous blood. Both SCINA and CellAssign require an input of gene signatures to annotate the query dataset. Users need to know what cells are potentially included in the query dataset and have some biological background about the cell-expressed genes. The two methods provide several gene lists, and we combine them and search through existing studies about marker genes to make our gene lists for annotation. We check the annotation per cell to calculate the accuracy.

In Figure 3, ‘Hard_enrich’, ‘soft_enrich’, ‘hard_fisher’ and ‘soft_fisher’ correspond to the method using the GSEA approach with hard classification, GSEA approach with soft classification, Fisher’s exact test approach with hard classification and Fisher’s exact test approach with soft classification.

Overall, the hard fisher classifier has similar performance as scCATCH, CellAssign, SCINA and SCSA. During our exploration, we find that SCSA focuses more on major lineage (e.g. T cell). Our dataset contains more sub-cell-type information (e.g. CD4 T helper cells, memory T cells, native cytotoxic T cells, naïve T cells and regulatory T cells) and SCSA cannot distinguish them well. As the cell number increases, the performance also becomes better. The accuracy of hard enrich is higher than that of CellAssign and SCINA. It is also higher than the results using SCSA and scCATCH when the query dataset contains 2400 and 1600 cells. When the cell number increases to 4000, the hard enrich classifier outperforms the other four annotation tools. Moreover, we compare the positive predictive values of the proposed hard classifiers and the four tools and present the results in Supplementary Figure S3. The precision values are not stable for different cell types. For example, most of the methods are good at identifying cytotoxic T cells and monocytes. Only scCATCH and hard enrich can identify regulatory T cells. CellAssign and hard-fisher demonstrate large variances of PPVs in identifying memory T cells, while other methods failed to identify this cell type.

For CellAssign, scCATCH and SCINA, the annotation accuracy can also be influenced by the choices of gene signatures. We performed additional simulation studies by extracting marker gene information from the CellMarker database, and using it as signature genes when applying scCATCH, CellAssign and SCINA. The markers are from the human species and blood-related tissues. Supplementary Figure S4 shows the application results over 20 Monte Carlo simulations. Our proposed methods demonstrate comparable or better results than existing methods. ScCATCH has the best performance among all the existing tools.

To precisely evaluate the results from soft classification, we include a separate figure with the results by soft classifications in Figure 3D. We consider the top two, three and five cell-type predictions in this evaluation. Overall, the GSEA method outperforms the Fisher’s exact test methods. It should be noted that the false positives exist when using the soft classification, as it reports the top several cell types for each cluster.

In addition, we also compare our methods with supervised annotation tools, Garnett (Pliner *et al.*, 2019), scPred (Alquicira-Hernandez *et al.*, 2019) and SingleR (Aran *et al.*, 2019) through a series of simulations with a different number of cells. The results are presented in Supplementary Figure S5. For Garnett, we use a pre-trained classifier for PBMCs provided on its website. For scPred, we follow the tool instruction to train a classifier using 3500 cells sampled from our PBMCs dataset. For SingleR, we use the default reference ‘HumanPrimaryCellAtlasData’ provided by its package. We apply the three methods on datasets with a different number of cells. We check the annotation per cell to calculate the accuracy.

Garnett outputs two different types of annotation: one is annotated for each cell (‘Garnett-cell type’), and the other is annotated for a cluster (‘Garnett–cluster type’). scPred also provides two different annotations for users: one is annotated cell type (‘ScPred-prediction’), and the other is the cell type that cannot be rejected (‘ScPred-no rejection’). We include all of those different annotations to make the comparison fair. The accuracy is steady on datasets with different numbers of cells, although the supervised methods can perform a litter better when the number of cells increases. The supervised methods, except for Garnett and SingleR, generally have better performances than our hard classifiers, but comparable to or worse than our soft classifiers. Overall, our methods can provide users with helpful cell-type information if the supervised methods cannot be applied.

### 3.2 Impacts of different sample sizes

We conduct additional simulations to investigate how the proposed method can be impacted by different settings. First of all, we investigate impacts from difference sample sizes and clustering softwares. We sample datasets with a different number of cells. We select two unsupervised algorithms to cluster the data, Seurat (Satija *et al.*, 2015) and SC3 (Kiselev *et al.*, 2017). Both methods have been widely used to cluster cells from scRNA-seq data in previous studies (Butler *et al.*, 2018, Zhao *et al.*, 2020). SC3 adapts a consensus-based clustering while Seurat uses community-based cluster detection. As a result, Seurat is more computationally efficient than SC3. However, Seurat does not allow the specification of the cluster number as SC3 does. For SC3, we specify the number of clusters to be 8, the same as that in the original data. For Seurat, we fix the clustering precision parameter as 1. After obtaining the clustering labels, we identify the differentially expressed markers for each cluster by setting different log fold change thresholds in Seurat and different area under the ROC curve (AUROC) thresholds in SC3.

The accuracy of annotation with different numbers of cells used in the analysis is showed in Supplementary Figure S6A and B. Supplementary Figure S6A uses SC3 for clustering while Supplementary Figure S6B uses Seurat.

First, we observe that soft classification has higher accuracy than hard classification in all the results. This is expected since soft classification generates more labels to compare with the true labels than hard classification. Second, although the soft classification using Fisher’s exact test has slightly higher accuracy compared with GSEA in Supplementary Figure S6A, overall we find the GSEA method achieves comparable or better performance for both soft and hard classification. Third, the increase of cell numbers improves the annotation accuracy for both panels A and B, but the improvement is quite small no matter which annotation approach we use or what the sample size is. This suggests that the proposal is reliable even with a small dataset of fewer than 2000 cells in total and eight cell types. Lastly, we do not observe significant improvement in the annotation accuracy when the correct number of clusters is specified using SC3. It is possible that the correct specification of cluster number does not solve the difficulty of annotating a few highly correlated cell types, and thus SC3 does not provide a significant advantage over Seurat in the current settings.

### 3.3 Impact of different cutoffs

Different cutoffs can be selected when identifying the cell-type-specific differential genes. Choosing a loose or stringent cutoff will result in a longer or shorter differential marker lists. We ask how these cutoffs can impact the annotation accuracy. In the experiments of this section, we fixed the number of cells as 2400 in all scenarios. Supplementary Figure S6C and D presents the accuracy of cell-type annotation under different cutoffs for AUROC and log fold change using marker genes found by SC3 and Seurat, respectively.

We find that overall the modified Fisher’s exact test with soft classification has the most stable performance with different levels of cutoff. Improvements of the annotation accuracy are observed when decreasing the cutoffs for log fold change or AUROC, especially when GSEA is selected. In Supplementary Figure S6D, When the log2 fold change threshold is too conservative (0.5), fewer genes are detected and the accuracy of the annotations is low. When the log2 fold change decreases, the accuracy will increase but the increase will stop at a certain length. We believe the annotation result is sufficiently good when FDR is 0.15. If the length of the marker gene list is too short, we suggest that the users may consider including more samples or merging a few similar clusters to obtain longer marker lists. Further examinations are also needed to confirm the validity of the annotations. Overall, a looser criterion will result in better accuracy.

The cell-type-specific markers are identified for each cell type by comparing it versus all the other cell types. We have tried several simulations using different tests when finding the DE genes with Seurat: DESeq2, Wilcoxon and *t*-test. The accuracy of annotation results is shown in Supplementary Figure S7. Overall, we suggest not to choose *t*-test when detecting DE genes. We also observe that the GSEA method works better with DESeq2. When the number of cells is less, Fisher’s exact test also favors DESeq2 a little.

### 3.4 Impact of different databases

The accuracy results using different databases are presented in Supplementary Figure S8. We draw 2400 PBMCs and annotate them using the three databases separately. When database from clustermole was chosen, we specified tissue types as blood, blood vessel and immune system. When PanglaoDB was chosen, we specified tissue types as immune system. After filtering, CellMarker had the most marker genes which is over 3000, while PanglaoDB had the fewest marker genes which are around 1000. We find that overall CellMarker database achieves the highest annotation accuracy, especially when GSEA is chosen. Probably due to the limited number of maker genes, using PanglaoDB results in the lowest accuracies for both GSEA and the modified Fisher’s exact test.

Additionally, our simulation results demonstrate the importance of specifying tissue type, as specifying tissue type can greatly improve the outcome accuracy compared to the analysis without such specification (Supplementary Fig. S9). In summary, our method can provide accurate information for researchers to assign annotation for scRNA-seq data.

## 4 Real-data analysis

### 4.1 Application on autism spectrum disorder data

Autism spectrum disorder (ASD) refers to a neurological and developmental disorder including a variety of disabilities in learning, communication and other social behaviors. Gene expression studies have revealed the changes in the neocortex of autism patients. We obtain an ASD dataset from a study that uses scRNA-seq to identify autism-associated transcriptomic changes in specific cell types (Velmeshev *et al.*, 2019). According to the original publication, tissue samples were collected from 15 ASD patients and 16 controls that are matched on age, sex, RNA integrity number and postmortem interval. After preprocessing with the 10X Genomics CellRanger platform, unbiased clustering of nuclear profiles from ASD and control samples was performed using the expression data. The clusters were then annotated according to the expression of known cell-type markers by the authors. The dataset is publicly available on the UCSC Cell Browser at https://cells.ucsc.edu/?ds=autism. It contains 104 559 cells from 11 neuronal and 6 glial cell types, including subtypes of excitatory neurons and interneurons, the main types of glial cells and brain endothelial cells. Since our purpose is to evaluate the marker-based annotation using the proposed method, we use the clustering assignment from the original data and generate the cluster-specific differential markers using Seurat. We set the cutoff of log fold change to be 0.25 when identifying expressed markers. Finally, around 460 marker genes are detected for each cluster.

**Fig. 4.**
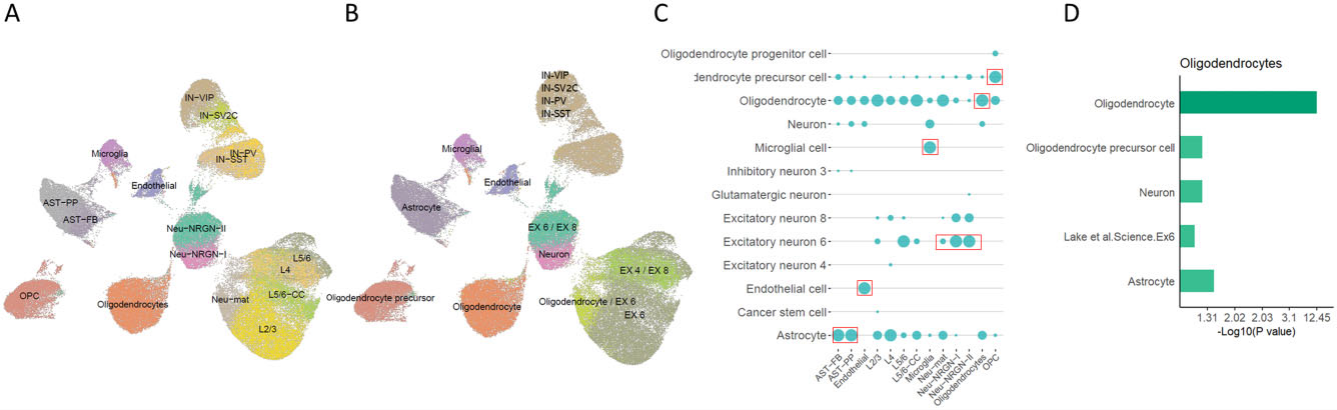
Results of cell-type annotation using GSEA approach for the ASD scRNA-seq data. (**A**) TSNE plot showing the true cell type for each cluster. (**B**) TSNE plot showing the predicted cell type for each cluster. (**C**) Dot plot showing the possible cell types for each cluster. X-axis shows the clusters we have, and Y-axis shows the candidate cell types. In each cluster, the significance of candidate cell type is presented by the size of the corresponding dot: the most significant cell type has the largest dot. Correct annotations are noted in the rectangle. (**D**) An example bar plot showing the possible cell types for cluster ‘Oligodendrocyte’. The cell type identified by hard classification is colored in dark green. Cell types in light green are identified by soft classification. Cell types from most significant to least significant are presented by the length of the bar

We use the CellMarker database for the annotation analysis. Tissues that are containing keyword ‘brain’ are selected, which are brain, embryonic brain, midbrain and fetal brain. We remove cells which belong to IN-VIP, IN-SV2C, IN-PV or IN-SST, because these four cell types are not included in the CellMarker database, and we have difficulty identifying similar terms. Figure 4 presents the annotation results from applying the GSEA approach.

Figure 4A shows the true cluster labels for all the cells. Figure 4B demonstrates the most possible cell-type assignment using our package. Comparing the two figures, we see that clusters ‘ASR-FB’, ‘AST-PP’, ‘Endothelial’, ‘Microglia’, ‘Oligodendrocytes’ and ‘OPC’ in the original ASD dataset are correctly annotated. For the other clusters, the candidate cell types are among Excitatory neurons and Inhibitory neurons, which are the subtypes of neurons. These cell types are consistent with the true labels from the original study.

Figure 4C summarizes the annotation results using the proposed method. The X-axis shows the clusters we have, and the Y-axis shows the candidate cell types. We present the top five significant cell types in each cluster. The significance of candidate cell types is presented by the size of the corresponding dot. The most significant cell type has the largest dot. We highlight cell labels that are consistent with the true cluster label using red rectangles. Although in several clusters, such as L4 and L2/3, the most significant cell does not match the true label, consistent cell types are still identified with less significance. In addition to the dot plot, for each cluster, we also generate a bar plot with up to 10 candidate cell types, and an example is given in Figure 4D. This bar plot shows the candidate cell types for cluster Oligodendrocyte. The significance of candidate cell types is represented by the length of the bar. We see that within this cluster the most significant cell type identified is Oligodendrocyte, followed by Astrocyte, Oligodendrocyte precursor cell and Neuron. In summary, our method accurately identifies the cell types for most of the clusters and provides related cell types for the other clusters.

### 4.2 Application on triple-negative breast cancer data

Lastly, we demonstrate the usage of our method in annotating a triple-negative breast cancer (TNBC) scRNA-seq dataset (Wu *et al.*, 2021a). According to the original study, the authors performed scRNA-seq (Chromium, 10X Genomics) on their samples, and clusters were annotated by canonical lineage markers. The authors then checked the annotation using published gene signatures to confirm its reliability. This dataset contains 10 836 tumor cells and 31 676 normal cells, including B cells, cancer-associated fibroblasts (CAFs), cancer epithelial, endothelial, myeloid, normal epithelial, plasmablasts, periventricular leukomalacia (PVL) and T cells.

For cancer-associated scRNA-seq studies, researchers usually separate the cancer cells from non-malignant cells before further annotation with existing tools, such as CopyKAT and inferCNV (Durante *et al.*, 2020, Gao *et al.*, 2021). Thus, we remove tumor cells from our dataset and conduct annotation on the normal cells only.

We applied three annotation tools on the dataset: The proposed method, SingleR and scCATCH. SingleR uses a rank-based correlation as a similarity metric. Our method also makes use of ranking information, and we would like to see if rank ordering of marker genes can help on the annotation when data are of a different phenotype compared to the reference. For the proposed method, we follow the analysis workflow as we did on ASD dataset in Section 4.1. We use Seurat for dimension reduction and the cluster assignment from the original paper as the cluster results. We identify the differentially expressed marker genes in each cluster. The cutoff for log fold change is still set to 0.25. Tissue types are specified as blood immune and breast related. Finally, around 700 marker genes are detected for each cluster. For SingleR, we use its built-in reference ‘HumanPrimaryCellAtlasData’. For scCATCH, tissue types are specified as blood and breast related.

**Fig. 5.**
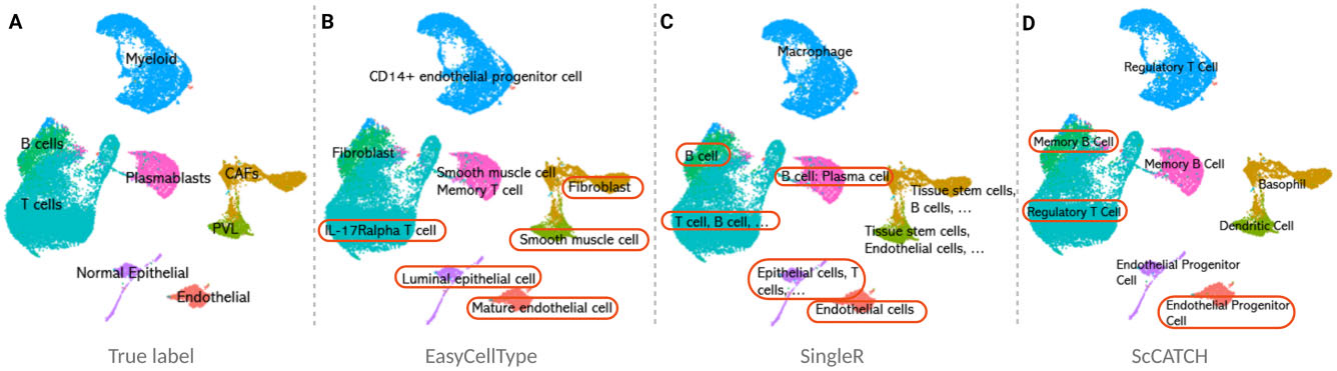
Results of cell-type annotation for the TNBC scRNA-seq data using different annotation tools. (**A**) True labels for each cluster. (**B**) Annotation results for each cluster using our proposed method. (**C**) Annotation results for each cluster using SingleR. (**D**) Annotation results for each cluster using scCATCH

The annotation results for each cluster are presented in Figure 5, where correct annotations are highlighted in red circles. For PVL cells, according to NCBI, perivascular cells include mainly vascular smooth muscle cells (SMCs) and pericytes. CAFs, T cell, PVL, epithelial and endothelial cells are annotated correctly by our method. For myeloid, a previous study (Bailey *et al.*, 2006) showed that endothelial cells are a component of differentiation of myeloid lineage. Our method might be limited when identifying the differentiation cells from a lineage. We observe that our method and SingleR performed slightly better compared to scCATCH, although SingleR generates cell types for each cell, resulting in impure annotations within the clusters.

## 5 Discussion

In this work, we develop a computational method to automatically examine the existing cell marker databases and annotate the cell-type labels with the marker information in a statistically rigorous way. As a crucial step in scRNA-seq analysis, many methods have been developed for annotating cells using supervised and unsupervised approaches. A recent comprehensive review of these annotating tools showed that, if matched training data are available, supervised methods generally achieve the highest accuracy for cell label assignment (Sun *et al.*, 2022). In this work, it is not our intention to outperform the supervised annotation methods but to provide a relatively accurate alternative for researchers to annotate cells when matched training data are not available and supervised methods cannot be directly applied. Additionally, when tissues from a novel condition (e.g. from diseased patients) are of interest, cluster-specific marker-based investigation is often conducted for a preliminary understanding of the tissue samples. This step can be challenging for researchers who are not biologically knowledgeable or lack domain expertise. Our method with three databases implemented will be a particularly helpful tool in these scenarios. Our implementations provide a graphical illustration of the annotated results including both dot plot summarization across all the clusters and the cluster-specific results with ranked bar plots. For users with R coding experiences, they can directly use our R/Bioconductor package, while for users with less coding experience, we recommend our shiny app with user-friendly graphical interface. We believe that the EasyCellType implementation can reduce the burden of annotating scRNA-seq data and provide useful guidance to achieve the most interpretable results.

Our method may be limited under certain situations. When one or several clusters contain mixed cell types, the annotation becomes harder as the cluster-specific markers will be mixtures from multiple cell types. In our simulation study, we find that some clusters contain multiple cell types. As the accuracy is computed per cell, these mixture clusters tend to have lower accuracy compared to other clusters. We acknowledge that this is a challenge shared by most of the annotation methods. Additionally, for the situation where cells are obtained from continuous cell differentiation, we believe that the clustering analysis and our proposed method may not fit well. Trajectory-based analyses such as Monocle (Trapnell *et al.*, 2014) and TSCAN (Ji and Ji, 2016) are better choices.

Lastly, we acknowledge that more databases are still under development and we plan to update our tool as they become available. As shown in our TNBC data application, the existing databases may not work well for specialized annotation in certain research areas such as cancer. Future work is needed to include more domain-specific knowledge to address these challenges.

## Supplementary Material

vbad029_Supplementary_Data

## Data Availability

The ASD data used in this article is available through https://cells.ucsc.edu/?ds=autism. The TNBC data used in the article is available through the GEO Series accession number GSE176078. The PBMC data used in the article can be downloaded in the 10X platform https://www.10xgenomics.com/cn/resources/datasets?query=pbmc&page=1&configure%5Bfacets%5D%5B0%5D=chemistryVersionAndThroughput&configure%5Bfacets%5D%5B1%5D=pipeline.version&configure%5BhitsPerPage%5D=500&configure%5BmaxValuesPerFacet%5D=1000. We also include the necessary information in the paper.
