# Supplementary material for: EasyCellType: marker-based cell-type annotation by automatically querying multiple databases
Source: Bioinform Adv. 2023 Mar 24;3(1):vbad029. doi: 10.1093/bioadv/vbad029 (PMC10049754; doi:10.1093/bioadv/vbad029)
Supplement: vbad029_Supplementary_Data [file vbad029_supplementary_data.pdf]

EasyCellType: Marker based cell type annotation by automatically querying multiple databases
Supplementary materials

Ruoxing Li[1,3], Jianjun Zhang[2], and Ziyi Li[3,*]

[1]Department of Biostatistics and Data Science, The University of Texas Health Science Center at Houston, Houston, Texas, 77030
[2]Department of Thoracic-Health & Neck Med Oncology, Division of Cancer Medicine, The University of Texas MD Anderson Cancer Center, Houston, Texas, 77030
[3]Department of Biostatistics, The University of Texas MD Anderson Cancer Center, Houston, Texas, 77030.
[*]Correspondance: zli16@mdanderson.org

GSEA

Suppose we have a set of genes sorted by their correlation with a phenotype of interest, and denote this gene set by L. Given a predefined gene list S, walking down L, a cumulative sum is calculated increasing by one if a gene of L in the predefined gene list and decreasing by one if not. Let g_j present the j_{th} gene in the sorted list L . Let r_j represent the correlation between the phenotype of interest and g_j. For g_j in S, we define:

$$P_{hit}(S,i) = \Sigma_{g_j \in S, j \leq i} \frac{|r_j|^p}{\Sigma_{g_j \in S}|r_j|^p}. \tag{1}$$

For g_j not in S, we define:

$$P_{miss}(S,i) = \Sigma_{g_j \notin S, j \leq i} \frac{1}{\Sigma_{N-N_H}}, \tag{2}$$

where p is a weight component, and i is a given position in L. Enrichment score is the maximum deviation from zero of $P_{hit} - P_{miss}$. Permuting phenotype labels 1000 times and calculating the enrichment score at eachtime, the null distribution of enrichment score can be obtained. P value for S is estimated by comparing the actual enrichment score to the null distribution. [3]

Exploration of cell marker databases

We explore the cell types and tissues (organs) in the 3 built-in cell marker data bases: Clustermole[1], PanglaoDB[2], and CellMarker[4]. Figures S1 to S2 present how cell types and tissues are intersected. We see that database from Clustermole contains most human cell types. No big difference is found regarding the number of Mouse cell types among the 3 databases. Clustermole contains some unique cell types such as Abomasum and Krt4/13+ compared to the other two databases. We find that the CellMarker database generally has a more detailed classification, while PanglaoDB has a broader lineage classification.

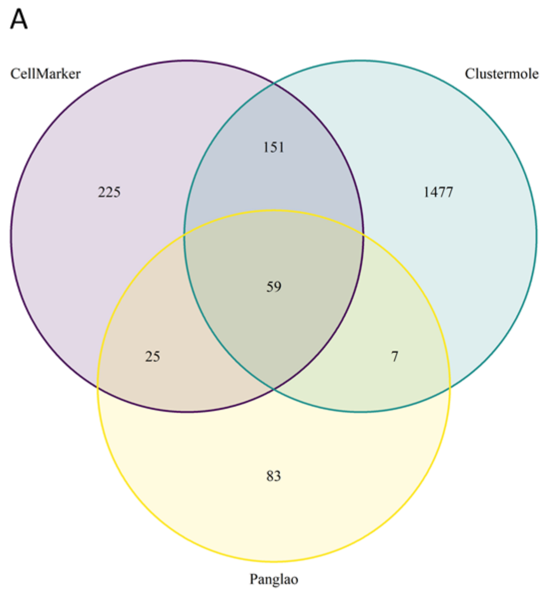

Figure S1: Number of cell types in CellMarker, PanglaoDB and Clustermole databases. (A) Number of Human cell types in CellMarker, PanglaoDB and Clustermole databases. The Clustermole has the most of cells. (B) Number of Mouse cells in CellMarker, PanglaoDB and Clustermole databases.

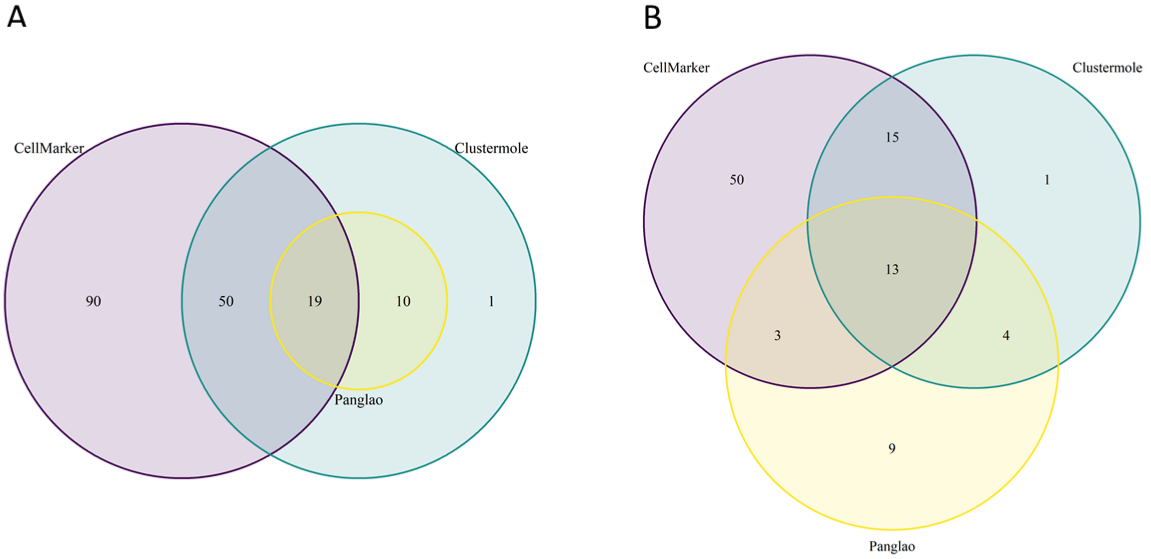

Figure S2: Number of tissues in CellMarker, PanglaoDB and Clustermole databases. (A) Number of tissues of Human in CellMarker, PanglaoDB and Clustermole databases. (B) NUmber of tissues of Mouse in CellMarker, PanglaoDB and Clustermole databases.

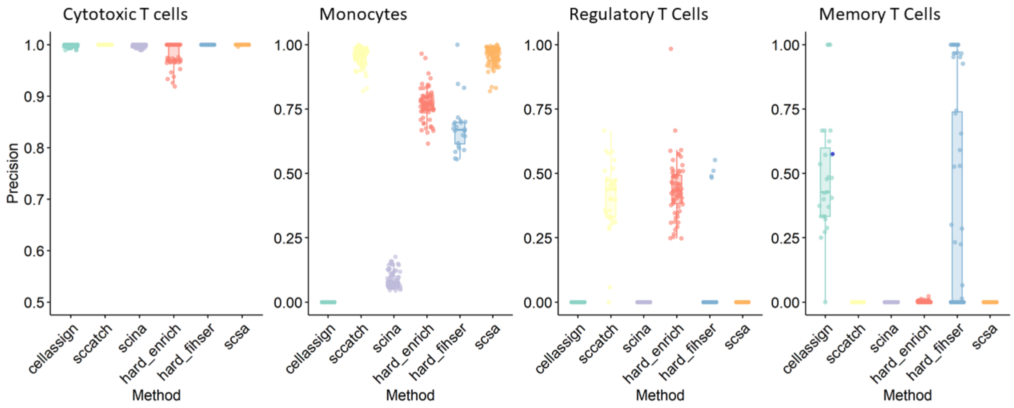

Figure S3: Positive predictive values of different cell types getting from the proposed hard classifiers and existing tools.

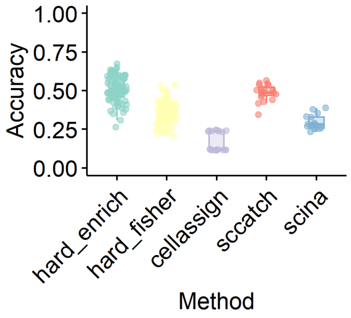

Figure S4: Annotation results using markers extracted from CellMarker database for CellAssign, scCATCH and SCINA.

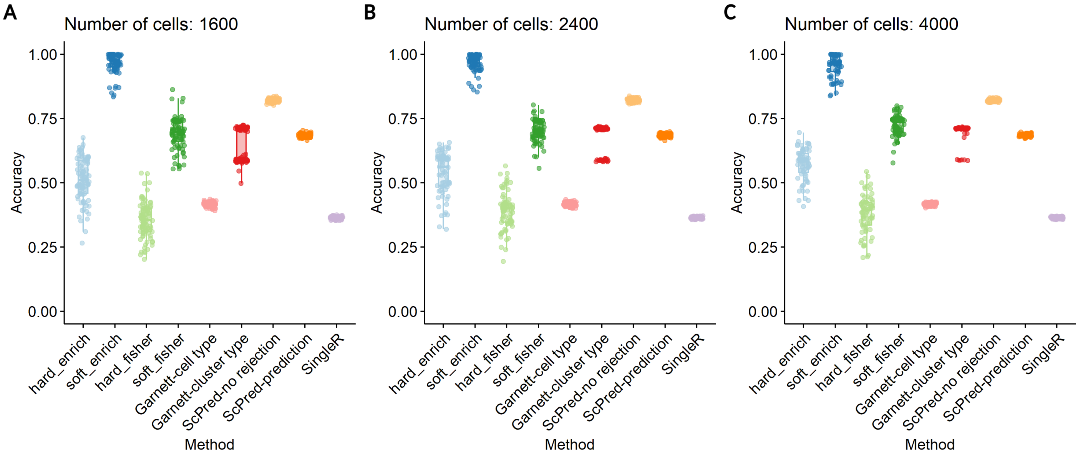

Figure S5: Accuracy of cell-type annotation under different cell number settings using the proposed method, Garnett, ScPred and SingleR. "hard_enrich" means that we use GSEA approach and hard classification. The same patterns are followed by other notations on the X-axis. (A) Sample data sets contained 1600 PBMCs. (B) Sample data sets contained 2400 PBMCs. (C) Sample data sets contained 4000 PBMCs.

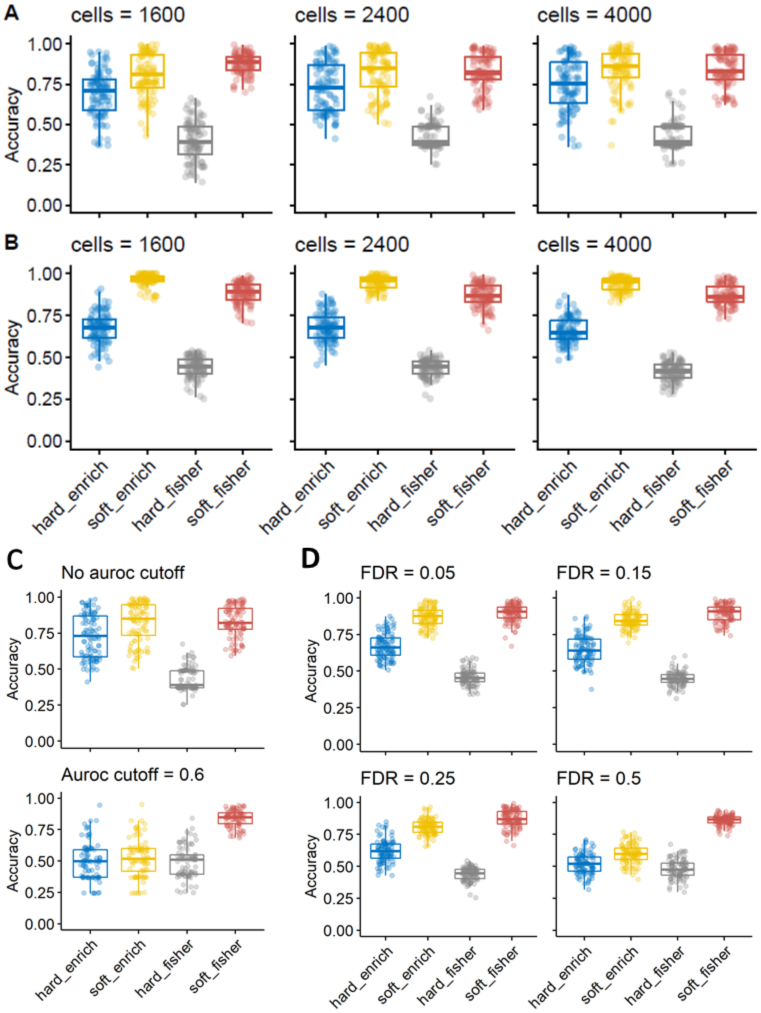

Figure S6: Accuracy of cell-type annotation under different cell number settings, and cutoff for AUROC and log fold change. “hard_enrich” means that we use GSEA approach and hard classification. The same patterns are followed by other notations on the X-axis. (A) Accuracy of cell-type annotation using differential makers analyzed by SC3. Sample data sets contained 1600, 2400, and 4000 PBMCs. AUROC cutoff was eliminated. The cutoff of P value of GSEA was set to 0.5. Blood-related tissues were specified. (B) Accuracy of cell-type annotation using differential makers analyzed by Seurat. Sample data sets contained 1600, 2400, and 4000 PBMCs. FDR cutoff was set to 0.25. P-value cutoff of GSEA was set to 0.3. (C) Accuracy of cell-type annotation using differential makers analyzed by SC3. The sample data set contains 2400 PBMCs. AUROC cutoff was eliminated or set to be 0.6. Cutoff of P-value of GSEA was set to 0.5. (D) Accuracy of cell-type annotation using differential makers analyzed by Seurat. The sample data set contains 2400 PBMCs. FDR cutoff was set to 0.05, 0.15, 0.25 and 0.5. The cutoff of P-value of GSEA was set 0.3.

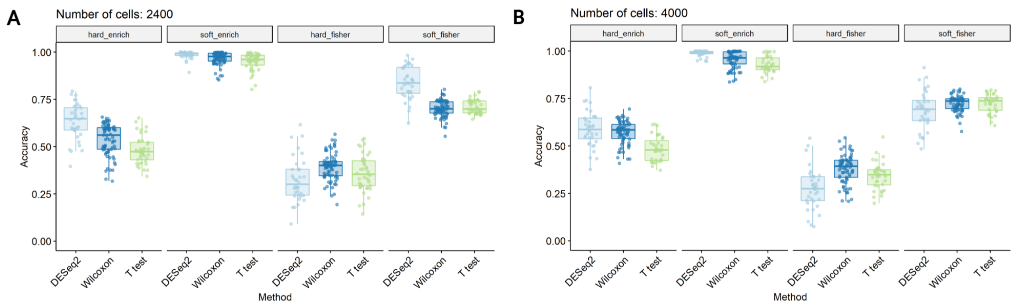

Figure S7: Accuracy of cell-type annotation using different methods that find differentially expressed genes with Seurat. (A) Sample data sets contained 2400 PBMCs. (B) Sample data sets contained 4000 PBMCs.

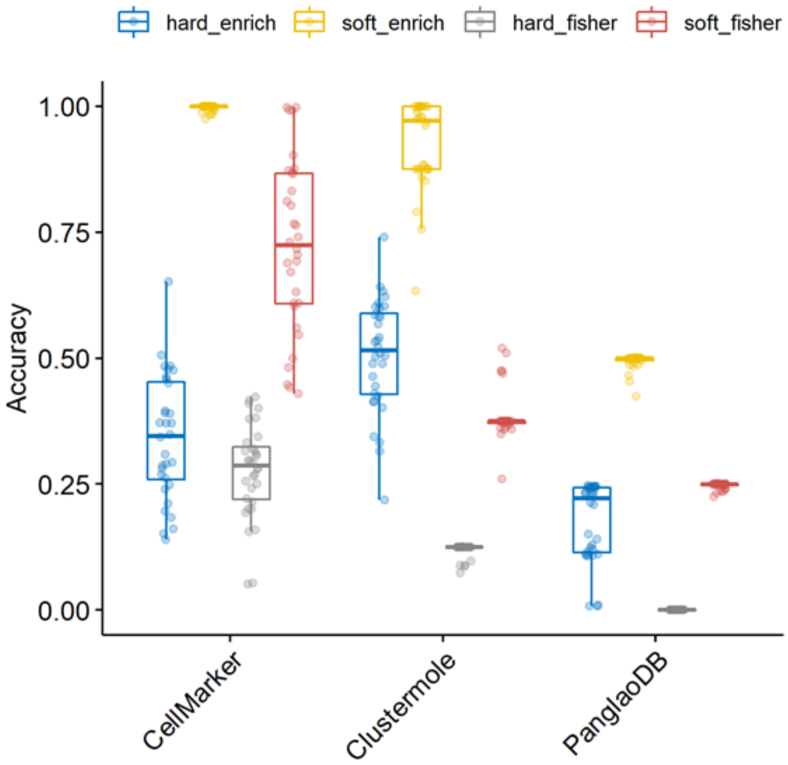

Figure S8: Accuracy of cell-type annotation using different databases. Sample data set contains 2400 PBMCs. "hard_enrich" means that we use GSEA approach and hard classification. The same patterns are followed by other notations in the figure legend. X-axis shows chosen databases. Y-axis shows annotation accuracy.

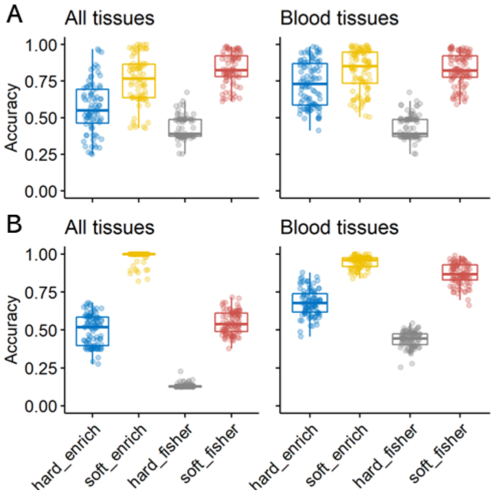

Figure S9: Accuracy of cell-type annotation under different tissue specification. "hard_enrich" means that we use GSEA approach and hard classification. The same patterns are followed by other notations on the X-axis. (A) Accuracy of cell-type annotation using differential makers analyzed by SC3. The sample data set contains 2400 PBMCs. AUROC cutoff was eliminated. Tissues are eliminated or specified as those related to blood. (B) Accuracy of cell-type annotation using differential makers analyzed by Seurat. The sample data set contains 2400 PBMCs. FDR cutoff was set to 0.25. Tissues are eliminated or specified as those related to blood.

References

[1] Igor Dolgalev. clustermole: Unbiased single-cell transcriptomic data cell type identification, 2021.

[2] Oscar Franzén, Li-Ming Gan, and Johan L M Björkegren. Panglaodb: a web server for exploration of mouse and human single-cell rna sequencing data. Database, 2019, 04 2019. baz046.

[3] Aravind Subramanian, Pablo Tamayo, Vamsi K. Mootha, Sayan Mukherjee, Benjamin L. Ebert, Michael A. Gillette, Amanda Paulovich, Scott L. Pomeroy, Todd R. Golub, Eric S. Lander, and Jill P. Mesirov. Gene set enrichment analysis: A knowledge-based approach for interpreting genome-wide expression profiles. Proceedings of the National Academy of Sciences, 102(43):15545–15550, 2005.

[4] Xinxin Zhang, Yujia Lan, Jinyuan Xu, Fei Quan, Erjie Zhao, Chunyu Deng, Tao Luo, Liwen Xu, Gaoming Liao, Min Yan, Yanyan Ping, Feng Li, Aiai Shi, Jing Bai, Tingting Zhao, Xia Li, and Yun Xiao. Cellmarker: a manually curated resource of cell markers in human and mouse. Nucleic Acids Research, 47(D1):D721–D728, 10 2018.
